# Dietary Use of *Hericium coralloides* for NAFLD Prevention

**DOI:** 10.3390/nu18030418

**Published:** 2026-01-27

**Authors:** Darya Chekushkina, Oksana Kozlova, Elena Vechtomova, Alexander Prosekov

**Affiliations:** 1 Genomics and DNA Sequencing Laboratory, Kemerovo State University, Kemerovo 650043, Russia; 2 Food Technology Institute, Kemerovo State University, Kemerovo 650043, Russia; ms.okvk@mail.ru (O.K.); vechtomowa.lena@yandex.ru (E.V.); 3 Laboratory of Biocatalysis, Kemerovo State University, Kemerovo 650043, Russia; a.prosekov@inbox.ru

**Keywords:** non-alcoholic fatty liver disease, preventative nutrition, *Hericium coralloides*, metabolites

## Abstract

*Introduction*: Today, scientists are searching for alternative approaches to preventing metabolic diseases, particularly non-alcoholic fatty liver disease, which reduces the healthy life expectancy of the working population. Fungi, such as *Hericium coralloides* (Scop.) Pers., are promising raw materials for extracting bioactive substances with preventative potential. *Materials and Methods*: This review covered review and research articles published over the last 42 years and indexed in the databases of the eLIBRARY.RU, the National Center for Biotechnology Information, and Scopus. *Results and Discussion*: It has been established that *H. coralloides* is valued for its nutritional properties due to its rich protein, fat, and mineral composition. It is in demand for pharmaceutical purposes due to its content of bioactive metabolites. The most studied metabolites are lovastatin and ergothioneine. The activity of these biologically active substances against NAFLD has been confirmed by studies in vitro and in vivo. Market analysis revealed that most dietary supplements contain fungal mycelium or its extract. It is preferable to use pure metabolites of *H. coralloides* as nutrients in dietary supplements and functional foods, since it allows the scientists to standardize their doses, target the therapeutic effect (immunity, neuroprotection, or antitumor), and reduce the required intake of the product. Since this fungus is a rare species in nature, its biomass should be grown in vitro for industrial use. *Conclusions*: Further research will focus on developing methods for extracting *H. coralloides* metabolites and assessing their biopotential in vivo and clinical studies.

## 1. Introduction

**Figure 1:**
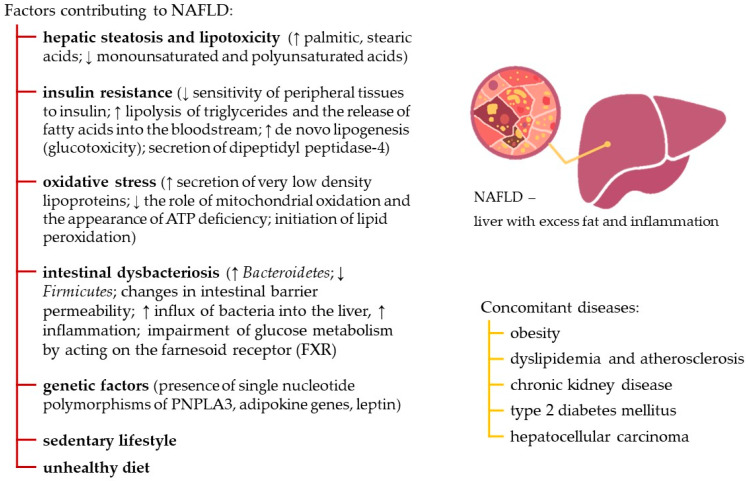
Factors contributing to NAFLD and concomitant diseases [2]: (↑)—increase; (↓)—decrease.

Non-alcoholic fatty liver disease (NAFLD) is a multifactorial disease characterized by complex multicomponent pathogenesis and a large number of associated conditions, Figure 1 [1].

NAFLD is characterized by excessive fat accumulation in hepatocytes without evidence of alcohol abuse. Significant factors contributing to the disease include lipid metabolism disorders, oxidative stress, inflammatory processes, intestinal microbiota imbalance, and nutritional disorders (high-calorie diets with excess saturated fats and refined carbohydrates), as well as a sedentary lifestyle [3]. Also important are metabolites of the gastrointestinal microbiota, which affect liver cells through the gut–liver axis [4].

In recent decades, NAFLD has become one of the most common chronic liver diseases worldwide. According to the latest epidemiological data, its prevalence reaches 25–30% of the population in developed countries and continues to grow due to the increasing incidence of obesity, metabolic syndrome [5], and type 2 diabetes mellitus [6,7].

Currently, conventional treatments for NAFLD include lifestyle modification (diet and exercise) and drug therapy aimed at reducing the risk factors such as hyperlipidemia and insulin resistance [8]. However, there is still a lack of effective and safe drugs for the treatment of NAFLD [9]. This necessitates the development of new effective approaches to prevention and therapy, including nutritional support in the form of nutritional agents and natural bioactive substances [10]. Recent studies have emphasized the role of natural compounds, primarily polysaccharides, antioxidants, and anti-inflammatory metabolites, in regulating lipid metabolism, reducing hepatocellular stress, and modulating intestinal microbiota [11].

The nutraceutical market has recently seen an active development of bioactive supplements and functional foods with preventative and therapeutic effects on NAFLD [12]. These products include polysaccharides, flavonoids, phytosterols, vitamins, and microelements that affect fat and glucose metabolism, as well as the state of the intestinal microbiota [13,14]. There is evidence that plant metabolites, such as resveratrol, exert a protective effect against NAFLD by modulating the intestinal microbiota. The study observed an improvement in the density of intercellular contacts of enterocytes reduces the translocation of lipopolysaccharides into the portal bloodstream and, accordingly, reduces the activation of the TLR4/NF-kB-dependent inflammatory cascade in the liver, which is considered as a key factor in the transition of steatosis to steatohepatitis [15]. Thus, the regulation of the “gut–liver” axis may be one of the most promising areas of application of polysaccharide fractions of *H. coralloides* in preventive nutrition. Of great importance are plant and fungal extracts rich in polyphenols, as well as microbial metabolites containing organic acids, amino acids, vitamins, and other bioactive substances [16]. These metabolites promote health by preventing metabolic syndrome and maintaining a healthy lifespan [15,17].

The fungus *Hericium coralloides* (Scop.) Pers., a member of the *Hericiaceae* family, is highly valuable. It contains a wealth of bioactive compounds, such as polysaccharides, hericenones, ericenes, and other metabolites with pronounced antioxidant, anti-inflammatory, and neuroprotective properties [18].

In experimental models, *Hericium* fungal polysaccharides demonstrate the ability to reduce the expression of lipogenic transcription factors (SREBP-1c, FAS) and activate the AMPK signaling pathway, which leads to a decrease in triglyceride accumulation in hepatocytes and an improvement in insulin sensitivity [19].

Despite the substantial body of research on *Hericium erinaceus*, data on *Hericium coralloides* remain fragmented and are mainly focused on its neurotrophic properties, whereas its potential in the context of metabolic liver diseases has not been systematically evaluated. Transcriptomic and chemical profiling studies indicate species-specific differences in secondary metabolite composition within the genus *Hericium*, including higher expression of terpenoid biosynthesis–related genes in *H. coralloides*, which may be relevant to the regulation of hepatic lipid metabolism and inflammatory responses. Therefore, the present review aims to bridge the gap between fundamental mycological research and applied nutraceutical approaches for the prevention of NAFLD [20].

Our aim was to conduct a systematic review of the scientific literature on the use of *Hericium coralloides* for the prevention of NAFLD. To achieve this aim, we set the following objectives:

(1) Review general information about *Hericium coralloides*;
(2) Examine the metabolic composition of the fungus, methods for its extraction, and extract yield;
(3) Analyze the market of dietary supplements containing *Hericium coralloides* (or its metabolites).

Thus, we sought to gather up-to-date information on the use of *Hericium coralloides* and determine the feasibility of using its metabolites to produce dietary supplements for preventing NAFLD.

## 2. Materials and Methods

The search covered review and research articles published in Russian and English over the last 42 years and indexed in the Russian Scientific Electronic Library (eLIBRARY.RU), the National Center for Biotechnology Information (NCBI) and Scopus (Elsevier). The market of products, which included *Hericium coralloides*, was studied, for which Internet resources were used.

The search keywords were “*Hericium coralloides* metabolites,” “*Hericium coralloides*-based dietary supplements,” “*Hericiaceae*,” “*Hericium coralloides*,” “metabolic diseases,” and “NAFLD prevention” (thematically related to the food industry, medicine, and biotechnology).

We focused on the papers describing the results of clinical and preclinical studies (regardless of the model objects), as well as review articles. We excluded the articles describing the results obtained in silico, conference proceedings, preprints and monographs.

**Figure 2:**
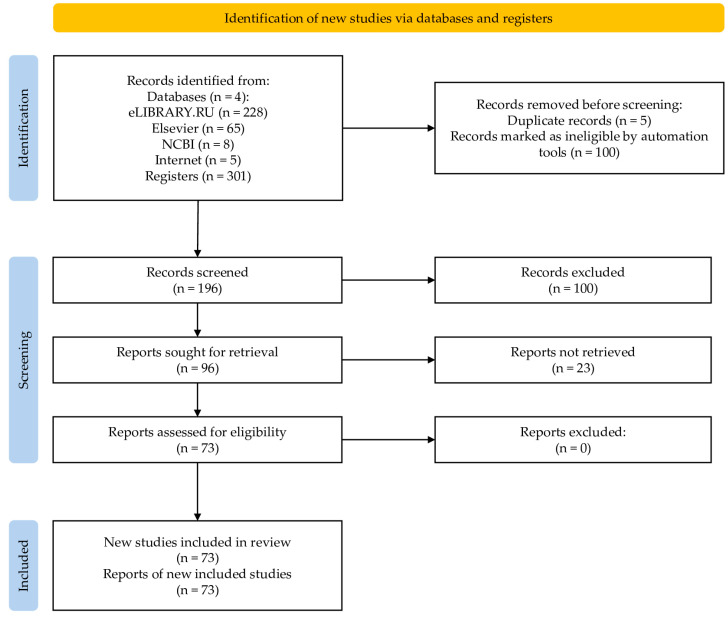
Preferred Reporting Items for Systematic Reviews and Meta-Analysis (PRISMA) flow chart of the study selection process [21].

The results of the literature search are shown in Figure 2.

## 3. Results and Discussion

**Figure 3:**
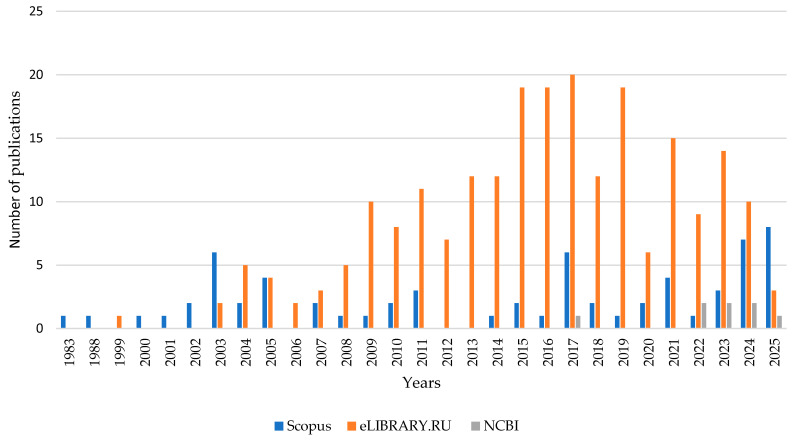
Number of publications for the keyword “*Hericium coralloides*” in databases by year.

The search of publications using the keyword “*Hericium coralloides*” showed a limited number of articles in the databases, Figure 3.

The first article on *H. coralloid* was published in 1983 [22].

The largest numbers of publications in Elsevier were 6 in 2003 (1983–2010), 6 in 2017 (2010–2020), and 8 in 2025 (2020–2025).

The largest numbers of publications in eLIBRARY.RU were 10 in 2009 (1983–2010), 20 in 2017 (2010–2020), and 15 in 2021 (2020–2025).

A total of 301 publications were returned by the search using the keyword “*Hericium coralloides*.” However, having screened all of them, we selected for this review 10 articles from eLIBRARY.RU, 9 articles from NCBI (excluding those also included in Elsevier), and 42 articles from Elsevier (excluding those also included in PubMed).

### 3.1. Information on Hericium coralloides

**Figure 4:**
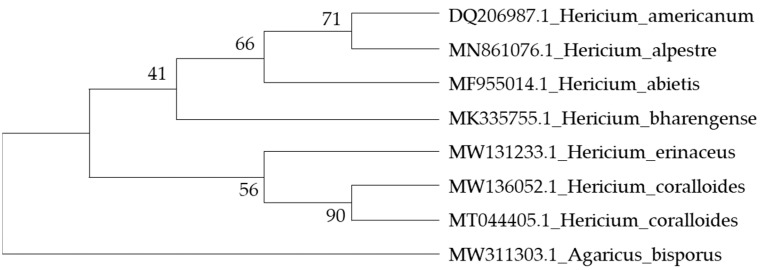
Phylogeny of *Hericium coralloides* reconstructed from the ITS dataset [23].

The genus *Hericium* belongs to the family *Hericiaceae*, the order *Russulales*, and the class *Agricomycetes*. Its 15 species are distinguished macroscopically by the presence of branched and unbranched hymenophore structures supporting spines of varying lengths, as well as by their occurrence in single or multiple clusters. Microscopically, they are distinguished by the presence of amyloid-ornamented basidiospores [23], Figure 4.

The species *Hericium coralloides* (Scop.) Pers., commonly known as the “coral tooth,” is a striking representative of this genus. Superficially resembling coral, the fungus has bright white coloration, a narrow base, and coral-like branches densely covered with spines. *H. coralloides* primarily grows on the trunks of broad-leaved trees or rotten wood in northeast, northwest, and southwest China [24]. This rare edible and medicinal mushroom is valued for its beneficial nutritional and pharmacological properties.

Recent years have seen a growing interest in using *H. coralloides* in the food industry and biotechnology. Importantly, its cultivation methods play a key role in determining the quality of the resulting product and its commercial potential [24].

There are two main methods of cultivating *H. coralloides*: in natural conditions, the forests where the fungus grows on wood, and in vitro. Both methods have their advantages and limitations, which determine the future use of the fungus’s fruiting bodies in research and industry. In nature, *Hericium coralloides* can be found on old and decaying deciduous trees in temperate climates. The fungus grows slowly and requires rather specific conditions in terms of humidity, temperature, and wood composition. The influence of these factors on the chemical profile and biological activity of the fungus’s fruiting bodies is largely determined by the ecosystem in which it grows [25]. Such growing conditions have limited scalability and can also expose the fungus to external factors, such as competition with other species and wood diseases. This limits the commercial use of *Hericium coralloides* grown in natural conditions. Moreover, due to the high demand for the fungus, it has become a rare species in some regions. In Russia, for example, it is listed in the Red Data Books of 46 constituents [26,27].

In vitro cultivation of *Hericium coralloides* significantly expands the possibilities for its industrial production with controlled quality. In laboratories, the fungus can be grown in sterile media at optimal temperatures and humidity, with its chemical composition thoroughly controlled. Researchers can improve the concentration of certain active substances, such as polysaccharides and phenolic compounds, which is important for their use in the pharmaceutical and food industries [28].

The fungi of the genus *Hericium* boast a diversity of structurally complex bioactive compounds both in their fruiting bodies and in the mycelium. However, their chemical profile can vary significantly under the influence of cultivation conditions. According to Guan Y. et al., the fruiting bodies of *H. coralloides* cultured in vitro contained about 33% polysaccharides, 16% crude protein, 12% total ash, 8% reducing sugars, 6% crude fat, 5% crude fiber, 3% total triterpenoids, 1% total saponins, 0.88% total flavonoids, and 0.5% total alkaloids. In addition, the total contents of sterols and phenols were found at 0.43% and 0.18%, respectively [27,29].

Nutritionally, *H. coralloides* has about 8–24% protein, a moderate fat content, and a rich mineral composition [30,31].

According to [32,33], the fungus’s genome was 43.7 Mb in size, consisted of 13 chromosomes, and had an N50 of 3.60 Mb. Its chromosome assembly efficiency was 96.9%. The transcriptor analysis revealed high expression of genes responsible for the terpenoid backbone and diterpenoid biosynthesis in the mycelium. In addition, the researchers identified the key genes involved in terpenoid biosynthesis. Their results also suggest the potential for commercial exploitation of *H. coralloides* terpenoid metabolites.

### 3.2. H. coralloides Metabolites, Methods of Their Extraction, and Extract Yield

Zhang et al. [34] aimed to optimize the extraction of polysaccharides from *Hericium coralloides* by comparing the efficiencies of various extraction methods. They employed both traditional and modern methods, including:

High-reflux extraction (HRE-P);
Acid-base extraction;
Enzymatic extraction;
Ultrasonic-assisted extraction (UAE-P);
Cold water extraction (CWE-P);
Pressurized hot water extraction (PHE-P).

The researchers also investigated hydrogen peroxide/ascorbic acid systems (HAE-P) and acid–chlorite delignification.

Their results demonstrated significant intergroup differences in extract yield, chemical composition, monosaccharide profile, and molecular weight. All nine polysaccharide fractions obtained showed similar characteristics when analyzed by Fourier transform infrared (FTIR) spectroscopy and nuclear magnetic resonance (NMR) spectroscopy. Among the methods, high-reflux extraction produced the second-highest polysaccharide yield, as well as the highest polyphenol content and the highest molecular weight of polysaccharides. The metabolites obtained by high-reflux extraction demonstrated the most pronounced antioxidant activity against ABTS (2,2′-azino-bis(3-ethylbenzothiazoline-6-sulfonic acid) and OH radicals. Cold water extraction revealed the highest antioxidant activity against ABTS, DPPH (2,2-diphenyl-1-picrylhydrazyl), and superoxide radicals, while ultrasonic-assisted extraction showed the best results against DPPH radicals [35].

Furthermore, the polysaccharides obtained by UAE-P, CWE-P, and HAE-P exhibited more pronounced cytoprotective activity against L929 cells compared to the other fractions. According to the correlation analysis, monosaccharide composition and total polyphenol content are key variables determining the bioactivity of *H. coralloides* polysaccharides [36].

Corallocins are a group of meroterpenoids that are benzofuranone and isoindolinone derivatives. Wittstein et al. [36] isolated three corallocins, namely A (2.8 mg), B (29.4 mg), and C (3.4 mg), from 7.88 g of crude ethyl acetate extract of *H. coralloides* fruiting bodies, whereas Ryu et al. [37] isolated corallocins from *H. erinaceus* fruiting bodies. Their chemical structures were examined by various spectral methods, including one-dimensional (1D) and two-dimensional (2D) nuclear magnetic resonance (NMR) spectroscopy, as well as high-resolution electrospray ionization mass spectrometry (HR-ESIMS) [37]. Subsequently, the same research group discovered two previously undescribed isoindolinone derivatives, which were named corallocin D and corallocin E. The crude ethyl acetate extract (272 mg) was purified by preparative reversed-phase liquid chromatography to isolate 1 mg of corallocin C and 0.7 mg of corallocin D.

The structures of these compounds were identified using HR-ESIMS and NMR analysis. The two compounds had a similar single chiral center and similar positive specific optical rotation values ([α]^20^D +24 and +26, respectively). Their absolute configuration was determined as an R-configuration by electron circular dichroism (ECD) [38].

The comparative structural analysis revealed that corallocins B–E have a common isoindolinone structure differing in the modification of the substituent at the nitrogen atom 2. Corallocin C, in turn, is a rare derivative of indoleisoindolinone. In contrast, corallocin A has a meroterpenoid structure of the benzofuranone type, with a carboxyl group located far from the geranyl fragment [23].

Studies have revealed significant neurotrophic potential of the compounds isolated from *Hericium coralloides*. Corallocins A, B, and C were examined for their ability to stimulate differentiation of PC12 cells (a rat pheochromocytoma cell line) by stimulating the astrocytoma cell line 1321N1 [36].

Corallocins A and C were found to increase the nerve growth factor (NGF) production in astrocytoma cells (line 1321N1) and stimulate neurite outgrowth in PC12 cells. Corallocins B and C also increased the expression of the brain-derived neurotrophic factor (BDNF) [33,37].

In another study, corallocin A isolated from *H. erinaceus* demonstrated its ability to induce strong survival and neurotrophic responses in cultured hippocampal neurons (DIV3) without the addition of serum [38]. Furthermore, corallocins D and E showed weak to moderate cytotoxicity against HeLa (KB 3.1), *Mucor hiemalis*, and *Bacillus subtilis* cells in vitro [39].

Lovastatin and ergothioneine (often referred to as the “longevity vitamins”) are valuable metabolites found in both the mycelia and fruiting bodies of *Hericium* fungi [40].

In [41], the scientists compared the contents of ergothioneine and lovastatin, as well as those of bioelements and glucan, in the fruiting bodies and mycelia of *H. erinaceus*, *H. coralloides* and *H. americanum*. They found that lovastatin was present in higher concentrations in the biomass obtained from the in vitro cultures compared to the fruiting bodies [23]. Among the three species, lovastatin had the highest content in the mycelium of *H. coralloides* (21.6 mg/100 g dry weight) and the lowest in that of *H. erinaceus* (5.81 mg/100 g dry weight). Unlike lovastatin, ergothioneine accumulated predominantly in the fruiting bodies of the fungi rather than in the in vitro cultures. In particular, its content was high in the fruiting bodies of *H. erinaceus* (305 mg/100 g dry weight) and lower in those of *H. coralloides* (155–177 mg/100 g dry weight) [41,42].

The presence of naturally occurring lovastatin in the biomass of *H. coralloides* adds further significance to this species as a potential source of hypolipidemic compounds. Inhibition of HMG-CoA reductase may contribute to reduced cholesterol biosynthesis and attenuation of systemic inflammation, which is clinically relevant in metabolic syndrome—a key risk factor for the development of NAFLD [43].

The main secondary metabolites of *Hericium* fungi are listed in Table 1.

The metabolites of *Hericium* fungi exhibit a wide range of biological activity. In particular, polysaccharides possess neuroprotective, immunomodulatory, and antioxidant properties, while erinacerins, hericerins, and other aromatic compounds exhibit neurotrophic and anticancer effects. Lovastatin and ergothioneine provide additional antioxidant, anti-inflammatory, and neuroprotective effects. Overall, the combination of these compounds makes the genus *Hericium* a promising source of bioactive substances for the development of functional foods and therapeutic agents [23].

In [33], the authors conducted a transcriptomic analysis to identify differential regulation of secondary metabolites of *H. coralloides* terpenoid fungi. The researchers used strain 77 of *H. coralloides* (Fujian, China). They found that this strain had dikaryotic hyphal morphology. The morphological analysis of its protoplasts (100× magnification) showed numerous clamp connections in the mycelium. These are mycelial bridges and anastomoses where fungal hyphae are tightly pressed or merge with each other, forming a stronger and more complex structure. These connections strengthen and support the mycelial strand, allowing the fungus to effectively transport nutrients throughout its network.

### 3.3. The Market of Dietary Supplements Containing Hericium coralloides (or Its Metabolites)

Dietary supplements containing *Hericium coralloides* and its metabolites lie at the intersection of nutraceuticals, functional mushrooms, and preventative medicine. Recent years have seen a growing interest in *Hericium* species as a source of compounds with neurotropic, anti-inflammatory, and metabolic-modulating properties [53]. Their potential hepatoprotective effects can help fight the increasing prevalence of non-alcoholic fatty liver disease (NAFLD). These effects of *Hericium* fungi are associated with their antioxidant activity, as well as their ability to reduce systemic inflammation and improve lipid and glucose metabolism.

The market of dietary supplements containing *Hericium coralloides* or its metabolites combines the interests in neurotropic, anti-inflammatory, and metabolically active natural compounds. These supplements are therefore considered as promising nutraceutical agents that can interact with the pathophysiological links of the disease and reduce the risk of its development in susceptible groups [54]. The products containing *Hericium coralloides,* which are available on the market, are presented in Table 2.

Most dietary supplements (Table 2) are based on dried and powdered *H. coralloides* mycelium or extract, rather than pure active metabolites. This leads to unstable concentrations of active ingredients and reduces the predictability of their effects. As a result, higher doses might be needed to achieve the desired result.

The analysis of the data collected during the literature review is reflected in Table 3.

The authors found no clinical studies confirming or refuting the effect of *H. coralloides* metabolites on NAFLD. They did find an in vivo study confirming the effect of ergothioneine on metabolic dysfunction-associated steatosis liver disease (MASLD) [71]. In the experiment, ergothioneine, administered daily at a dose of 35 mg/kg body weight to male mice C57BL/6J, improved lipid metabolism and reduced liver dysfunction in mice fed a high-fat diet.

The databases used in this study primarily include in vivo studies in rodents [46,66,72] and laying hens [19], which describe an indirect association with metabolic disorders, including NAFLD. In [19], the scientists studied the mechanisms by which polysaccharides isolated from *H. erinaceus* alleviate the symptoms of NAFLD in old laying hens, focusing on the regulatory function of the metabolites in the intestinal microbiome. They found that the polysaccharides ameliorated liver damage and metabolic disorders, as well as improved the intestinal barrier function. This reduced the transport of lipopolysaccharides from the intestine to the liver, inhibiting the activation of the hepatic pathway and slowing down the liver’s inflammatory response and apoptosis. Thus, *H. erinaceus* metabolites can mitigate liver damage and metabolic disorders associated with NAFLD by regulating the gut–liver axis [19].

These results demonstrate the value of further research examining the effects of *H. coralloides* metabolites on metabolic diseases in vitro, in vivo, and in humans. This literature review suggests that *H. coralloides* metabolites demonstrate potential for the prevention of NAFLD. Similar metabolites (atorvastatin, simvastatin) have also shown potential for the treatment of NAFLD in randomized, placebo-controlled trials [68].

Most of the data on the biological activity of *H. coralloides* metabolites were obtained in in vitro and experimental models of neurodegeneration, whereas direct models of NAFLD for this species are practically absent. Extrapolation of antioxidant and anti-inflammatory effects on liver tissue is possible only indirectly, through common pathogenetic mechanisms, including activation of the Nrf2 pathway, suppression of NF-kB, and decreased production of proinflammatory cytokines (TNF-α, IL-6), which play a key role in the progression of steatohepatitis. Similar mechanisms have previously been demonstrated for *H. erinaceus* polysaccharides in NAFLD models, which indicates the potential relevance of these pathways for *H. coralloides*, but requires direct experimental confirmation.

## 4. Conclusions

The data on *Hericium coralloides* metabolites suggest expanding the uses of this fungus as an ingredient in functional foods, nutraceuticals (dietary supplements), and cosmeceuticals due to its antioxidant properties. In addition to its traditional use as dried and powdered biomass in dietary supplements, the fungus’s individual bioactive metabolites (corallocins, erinacerins, and other neurotrophic compounds) could be introduced into functional foods and specialized dietary supplements. This will enable the scientists to standardize the composition of the products and increase their effectiveness, as well as to specifically implement their neuroprotective, immunomodulatory, and antioxidant effects for NAFLD prevention. The use of pure metabolites, or their concentrates, can open up new possibilities for developing products with proven pharmacological activity and more predictable and controllable biological effects compared to the traditional biomass [23].

Given the growing scientific interest in fungi of the genus *Hericium*, including the well-studied *Hericium erinaceus* [73], the biological potential of *H. coralloides* is relevant for both research and practical applications. Studying its composition, biological activity, cultivation methods, and technological applicability can contribute to expanding the range of functional ingredients and creating innovative nutraceutical products. This review covered the chemical composition, nutritional value, and biological activity of *H. coralloides*, as well as its potential use in the food industry, specifically in products supporting metabolic health and liver function.

Significant limitations of the current evidence base should be noted, including the lack of direct in vivo models of NAFLD using *H. coralloides* specifically, as well as the lack of clinical studies evaluating the effect of its metabolites on biochemical markers of liver function. Therefore, the use of this mushroom in nutraceutical products should be considered as a potentially preventive rather than therapeutic intervention. Future research will focus on developing standardized extracts with known levels of polysaccharides, ergothioneine, and terpenoids, using cell lines, animal models of NAFLD, and conducting randomized clinical trials evaluating biochemical and imaging markers of steatosis.

At the initial stage, in vitro studies will be conducted using cell lines relevant to the pathogenesis of NAFLD and metabolic disorders, including human hepatocyte cell lines (HepG2 and Huh7) and macrophage cell lines (THP-1) to evaluate anti-inflammatory activity. The investigations will focus on the regulation of genes involved in lipid metabolism (*PPARα*, *SREBP-1c*, *FAS*), oxidative stress (*Nrf2*, *SOD*, *CAT*), inflammatory responses (*NF-κB*, *TNF-α*, *IL-6*), and programmed cell death pathways, including apoptosis and autophagy (caspase-3). In addition, the effects of extracts and individual metabolites on mitochondrial function, reactive oxygen species levels, and cellular insulin sensitivity will be assessed. At the next stage, in vivo studies using animal models of NAFLD are considered appropriate, including diet-induced models (high-fat diet-fed mice or rats), which allow for the reproduction of key metabolic and morphological features of the disease.

Thus, the combination of studies on cell lines and validated animal models will enable the substantiation of the biological activity of *Hericium coralloides* at the level of molecular mechanisms and provide a scientific basis for the subsequent transition to clinical studies and the development of nutraceutical products with proven preventive potential against NAFLD and related metabolic disorders.

## Figures and Tables

**Table 1:** Secondary metabolites of fungi of the genus *Hericium*.

Fungus	Metabolite	Biological Activity	Model Object	Reference
*H. erinaceus*	Polysaccharide PHEB	Mitigating oxidative stress in the brain in Alzheimer’s disease by regulating Nrf2 and kinase; regulating calcium homeostasis in the brain; exhibiting anti-Alzheimer’s properties. PHEB polysaccharides ameliorate metabolic disorders (improving gut microbiota, reducing inflammation, and decreasing metabolic markers), which is associated with mechanisms relevant to NAFLD, including modulation of gut microbiota and regulation of metabolites, ultimately improving liver function via the gut–liver axis.	Laying hens	[19,44]
*H. erinaceus*	Heteropolysaccharides fraction HEP-W	Immunomodulatory activity.	Human cell lines	[45]
*H. erinaceus*	Polysaccharide HEFP	Antioxidant and anti-inflammatory activity; alleviating ulcerative colitis by inhibiting the NLRP3/caspase-1 inflammasome pathway.	Mouse	[46]
*H. coralloides*	Corallocins	Promoting the expression of nerve growth factor (NGF) in astrocytoma cells and neurite outgrowth in PC12 cells; stimulating the expression of brain-derived neurotrophic factor (BDNF).	Human cell lines	[36]
*H. erinaceus*	Erinacerins	Inhibiting effect on α-glycosidases (potential antidiabetic agents) and tyrosine phosphatase-1B (PTP1B); immunomodulatory and neuroprotective properties.	Human cell lines	[47]
*H. flagellum*	Hercioic and hericiofuranic acids	Neurotrophic activity.	Human and rat cell lines	[48]
*H. erinaceus*	Aromatic compounds: hericerin A, isohericenone J, isoericerin, hericerin, N-De phenylethyl isohericerin, hericenon J, 4-[3′,7′-dimethyl-2′,6′-octadienyl]-2-formyl-3-hydroxy-5-methoxybenzyl alcohol	Anticancer and neurotrophic activity.	Human cell lines	[43]
*H. erinaceus*	Erinacins	Neurotrophic activity; stimulating effect on the nerve growth factor (NGF)	Rat of the Wistar line	[49]
*H. erinaceus*, *H. coralloides*, *H. americanum*	Lovastatin	Anti-inflammatory, neuroprotective, and anticancer effects	Human cell lines	[50]
*H. erinaceus*, *H. coralloides*, *H. americanum*	Ergothioneine	Antioxidant, antiradiation, and anti-inflammatory activity	Review article	[51]
*H. alpestre*	Avenacin Y	Anticancer activity	Human cell lines	[52]

**Table 2:** Review of products containing *H. coralloides* (metabolites).

Product	Form	Active Ingredient	Biological Activity	Producer	Reference
Sorbiotic (dietary supplement)	Concentrate	*H. coralloides*	Detoxifying effect, restoring intestinal microflora	Art Life, Russia	[55]
Ezhovik korallovidny (dietary supplement)	Capsules	*H. coralloides* dried mycelium	Antioxidant, anti-inflammatory, and immunoregulatory activity	BIOFARM, Russia	[56]
Nativitan (dietary supplement)	Syrup	*H. coralloides*	Maintaining strong immunity and cognitive functions, increasing productivity; antitumor and anti-aging effects	Milfey, Germany	[57]
Fungosbor (dietary concentrate)	Capsules	*H. coralloides* extract	Stimulating effect in people with chronic fatigue	Batel, Russia	[58]
Ezhovik korallovidny + Ezhovik grebenchaty (dietary supplement)	Powder	*H. coralloides* mycelium	Neuroprotective, antiviral, immunomodulatory, and antitumor effects	Izviliny, Russia	[59]

**Table 3:** The potential of *Hericium coralloides* metabolites for the prevention of NAFLD.

Metabolite	Evidence Directly Related to NAFLD	Indirect or Supportive Metabolic Evidence	Hypothetical or Extrapolative Mechanisms
Polysaccharide PHEB	–	PHEB polysaccharides ameliorate metabolic disorders (including improvement of the gut microbiota and reductions in inflammation and metabolic markers), which is associated with mechanisms relevant to NAFLD, namely modulation of the gut microbiota and regulation of metabolites, leading to improved liver function via the gut–liver axis. The model object is laying hens [19]	Possible suppression of lipogenesis via AMPK activation and enhanced β-oxidation, as AMPK inhibits key lipogenic enzymes and promotes fatty acid oxidation. The model object is a human cell line [60]
Heteropolysaccharides fraction HEP-W	–	–	HEP-C from *Hericium erinaceus* ameliorated hyperglycemia and oxidative stress in diabetic rats, via activation of the PI3K/Akt signaling pathway, leading to improved glucose metabolism. The model object is a rat [60]
Polysaccharide HEFP	–	*–*	It enhances antioxidant defense and attenuates NF-κB–mediated inflammation, while AMPK can suppress NF-κB signaling and thereby reduce the inflammatory response. The model object is a mouse [61] Polysaccharides can reduce blood glucose levels and improve insulin sensitivity in diabetic models by increasing short-chain fatty acid (SCFA) levels and modulating immune-related signaling pathways, including PI3K/Akt and GLP-1. The model object is a mouse [62]
Corallocins	–	*–*	Antioxidant mechanisms, including the reduction of reactive oxygen species (ROS), may safeguard against oxidative stress, a key contributor to NAFLD pathogenesis by promoting mitochondrial dysfunction and inflammation. The model object is a human cell line [63]
Erinacerins	–	Compounds associated with antioxidant and anti-inflammatory effects may help improve systemic inflammation, which drives metabolic disorders, with similar effects observed in other models of inflammation. The model object is a mouse [46]	The anti-inflammatory effects may attenuate NF-κB–mediated cytokine production, thereby mitigating inflammatory processes that contribute to NAFLD progression. The model object is a rat [60]
Hercioic and hericiofuranic acids	–	Bioactive aromatic compounds derived from mushrooms have demonstrated antioxidant activity in in vitro studies, which may contribute to their metabolic effects relevant to NAFLD [64]	Antioxidant actions may strengthen the liver’s antioxidant system, reducing ROS-mediated damage and fibrosis. The model object is a human cell line [65]
Aromatic compounds: hericerin A, isohericenone J, isoericerin, hericerin, N-De phenylethyl isohericerin, hericenon J, 4-[3′,7′-dimethyl-2′,6′-octadienyl]-2-formyl-3-hydroxy-5-methoxybenzyl alcohol	–	–	Anticancer and neurotrophic activity. The model object is a human cell line [43]
Erinacins	–	Erinacines have been shown in high-fat diet–fed mice to reduce body weight, improve insulin resistance, and normalize blood glucose levels, indicating their potential in regulating metabolic disorders and obesity. The model object is a mouse [66]	Activation of AMPK can result in the downregulation of SREBP-1c and ACC, suppression of lipogenesis, promotion of β-oxidation, and stimulation of lipid droplet autophagy (lipophagy). The model object is a human cell line [67]
Lovastatin	Clinical and review literature is available on statins in NAFLD, including lovastatin as an HMG-CoA reductase inhibitor. The model object is a human [68]	Lovastatin reduces glucose uptake in cells, as observed in human cell cultures, where glucose transport into cells decreased following lovastatin treatment. This effect may indicate an influence on glucose metabolism and insulin-dependent processes. The model object is a human cell line [69]	Statins may modulate inflammatory pathways and NF-κB signaling, thereby reducing the progression of fibrosis. Model objects of mice and rats [70]
Ergothioneine	In a MASLD model, ergothioneine reduced hepatic steatosis and inflammation in vivo (mice). The model object is a mouse [71]	In NAFLD and MASLD models, ergothioneine has demonstrated antioxidant and anti-inflammatory activities, mitigating oxidative stress, enhancing lipid metabolic profiles, and preventing the worsening of metabolic outcomes. The model object is guinea pig cell lines [72]	Antioxidant activity can safeguard mitochondria from ROS-induced damage and attenuate inflammation, both of which are critical contributors to NAFLD progression. The model object is a human cell line [65]
Avenacin Y	–	*–*	Saponins can indirectly modulate the microbiota and barrier function of the intestine, affecting systemic inflammation and lipid metabolism associated with NAFLD. The model object is a human cell line [63]

## Data Availability

No new data were created or analyzed in this study. Data sharing is not applicable to this article.

## References

[1] Cheremukhina Y.V., Borovkova N.Y., Vasilkova A.S., Vlasova T.V., Akhmedzhanov N.M. (2025). Non-alcoholic fatty liver disease: A view formation on the problem and its significance for the clinical practice. Russ. J. Prev. Med..

[2] Kytikova O.Y., Novgorodtseva T.P., Denisenko Y.K., Kovalevsky D.A. (2020). Metabolic and Genetic Determinants of Lipid Metabolism Disruption in Non-Alcoholic Fatty Liver Disease. Russ. J. Gastroenterol. Hepatol. Coloproctol..

[3] Meroni M., Longo M., Rustichelli A., Dongiovanni P. (2020). Nutrition and Genetics in NAFLD: The Perfect Binomium. Int. J. Mol. Sci..

[4] Reshetova M.S., Zol’nikova O.Y., Ivashkin V.T., Ivashkin K.V., Appolonova S.A., Lapina T.L. (2022). Rol’ kishechnoj mik-robioty i ee metabolitov v patogeneze nealkogol’noj zhirovoj bolezni pecheni. Ross. Zhurnal Gastroentero-Logii Gepatologii Koloproktol..

[5] Chekushkina D.Y., Fedorova A.M., Kovalenko S.V., Milentyeva I.S., Altshuler O.G., Aksenova L.M. (2025). Anti-Metabolic Syndrome Effect of Trans-Cinnamic Acid. Food Process. Tech. Technol..

[6] Kosobyan E.P., Smirnova O.M. (2010). Modern concepts of pathogenesis of non-alcoholic fatty liver disease. Diabetes Mellit..

[7] Vesnina A., Le V., Ivanova S., Prosekov A. (2025). Antidiabetic Potential of Mangiferin: An In Silico and In Vivo Approach. Pharmaceutics.

[8] Chalasani N., Younossi Z., Lavine J.E., Charlton M., Cusi K., Rinella M., Harrison S.A., Brunt E.M., Sanyal A.J. (2018). The diagnosis and management of nonalcoholic fatty liver disease: Practice guidance from the American Association for the Study of Liver Diseases. Hepatology.

[9] Vesnina A.D., Frolova A.S., Chekushkina D.Y.u., Milentyeva I.S., Luzyanin S.L., Aksenova L.M. (2026). Gut microbiota and its role in development of chronic disease and aging. Foods Raw Mater..

[10] Prosekov A.Y., Vesnina A.D., Lyubimova N.A., Chekushkina D.Y., Mikhailova E.S. (2025). Consumer Genomics in Personalized Nutrition. Food Process. Tech. Technol..

[11] Yang S., Wei Z., Luo J., Wang X., Chen G., Guan X., She Z., Liu W., Tong Y., Liu H. (2024). Integrated bioinformatics and multiomics reveal Liupao tea extract alleviating NAFLD via regulating hepatic lipid metabolism and gut microbiota. Phytomed. Int. J. Phytother. Phytopharm..

[12] Poluektova E.A., Beniashvili A.G., Maslennikov R.V. (2020). Nutraceuticals and Pharmaceuticals. Russ. J. Gastro-Enterol. Hepatol. Coloproctol..

[13] Dangour A.D., Lock K., Hayter A., Aikenhead A., Allen E., Uauy R. (2010). Nutrition-related health effects of organic foods: A systematic review. Am. J. Clin. Nutr..

[14] Frolova A.S., Vesnina A.D., Fedorova A.M., Milentyeva I.S., Prosekov A.Y.u., Zaushintsena A.V. (2025). Hypoglycemic and hypocholesterolemic activities in vivo of polyphenols-popular components of dietary supplements. Nutr. Issues.

[15] Guo J., Wang P., Cui Y., Hu X., Chen F., Ma C. (2022). Alleviation Effects of Microbial Metabolites from Resveratrol on Non-Alcoholic Fatty Liver Disease. Foods.

[16] Bobrysheva T.N., Anisimov G.S., Zolotoreva M.S., Evdokimov I.A., Budkevich R.O., Muravyev A.K. (2025). Encapsulated polyphenols in functional food production. Foods Raw Mater..

[17] Uchendu I.K., Ikebunwa O.A., Okpagu C.B. (2024). Cardiorenal protective effects of extracts of bitter leaf (*Vernonia amygdalina* L.) in animal model of metabolic syndrome. Foods Raw Mater..

[18] Li G., Yu K., Li F., Xu K., Li J., He S., Cao S., Tan G. (2014). Anticancer potential of *Hericium erinaceus* extracts against human gastrointestinal cancers. J. Ethnopharmacol..

[19] Wu L., Hu Z., Lv Y., Ge C., Luo X., Zhan S., Huang W., Shen X., Yu D., Liu B. (2024). *Hericium erinaceus* polysaccharides ameliorate nonalcoholic fatty liver disease via gut microbiota and tryptophan metabolism regulation in an aged laying hen model. Int. J. Biol. Macromol..

[20] He X., Wang X., Fang J., Chang Y., Ning N., Guo H., Huang L., Huang X., Zhao Z. (2017). Structures, biological activities, and industrial applications of the polysaccharides from *Hericium erinaceus* (Lion’s Mane) mushroom: A review. Int. J. Biol. Macromol..

[21] Haddaway N.R., Page M.J., Pritchard C.C., McGuinness L.A. (2022). PRISMA2020: An R package and Shiny app for producing PRISMA 2020-compliant flow diagrams, with interactivity for optimised digital transparency and Open Synthesis. Campbell Syst. Rev..

[22] Bisht N.S., Harsh N.S.K. (1983). Relationship of lag phase duration to the texture and occurrence of wood-decaying fungi. Bull. Br. Mycol. Soc..

[23] Kostanda E., Musa S., Pereman I. (2024). Unveiling the Chemical Composition and Biofunctionality of *Hericium* spp. Fungi: A Comprehensive Overview. Int. J. Mol. Sci..

[24] Tabibzadeh F., Alvandi H., Hatamian-Zarmi A., Kalitukha L., Aghajani H., Ebrahimi-Hosseinzadeh B. (2024). Antioxidant activity and cytotoxicity of exopolysaccharide from mushroom *Hericium coralloides* in submerged fermentation. Biomass Convers. Biorefin..

[25] Pallua J.D., Recheis W., Pöder R., Pfaller K., Pezzei C., Hahn H., Huck-Pezzei V., Bittner L.K., Schaefer G., Steiner E. (2012). Morphological and tissue characterization of the medicinal fungus *Hericium coralloides* by a structural and molecular imaging platform. Analyst.

[26] Antonova L.D. (2019). Dva redkih vida gribov (*Hericium coralloides* i *Polyporus umbellatus*) na territorii Glavnogo botaniche-skogo sada Rossijskoj akademii nauk. Byulleten’ Mosk. Obs. Ispyt. Prir. Otd. Biol..

[27] Triskiba S.D. (2022). A new find of the fungus *Hericium coralloides* (Fr.) Pers. (Basidiomycota: Agaricomycetes: Hericiaceae) on the Donetsk Upland. Promyshlennaya Bot..

[28] Kumar H., Bhardwaj K., Sharma R., Nepovimova E., Cruz-Martins N., Dhanjal D.S., Singh R., Chopra C., Verma R., Abd-Elsalam K.A. (2021). Potential Usage of Edible Mushrooms and Their Residues to Retrieve Valuable Supplies for Industrial Applications. J. Fungi.

[29] Guan Y., Shi D., Wang S., Sun Y., Song W., Liu S., Wang C. (2023). *Hericium coralloides* Ameliorates Alzheimer’s Disease Pathologies and Cognitive Disorders by Activating Nrf2 Signaling and Regulating Gut Microbiota. Nutrients.

[30] Kumla J., Thangrongthong S., Kaewnunta A., Suwannarach N. (2025). Research advances in fungal polysaccharides: Production, extraction, characterization, properties, and their multifaceted applications. Front. Cell. Infect. Microbiol..

[31] Rupcic Z., Rascher M., Kanaki S., Köster R.W., Stadler M., Wittstein K. (2018). Two New Cyathane Diterpenoids from Mycelial Cultures of the Medicinal Mushroom *Hericium erinaceus* and the Rare Species, *Hericium flagellum*. Int. J. Mol. Sci..

[32] Li Y., Cai J., Li X., Hu X., Zhang J., Wu X., Fu J. (2025). Domestication Cultivation and Nutritional Analysis of *Hericium coralloides*. J. Fungi.

[33] Meng K., Lv J., Zhang T., Liu Y., Zhang P., Zhang Y., Hu B., Huang Q., Xie B., Fu J. (2024). Chromosome-Scale Genome and Transcriptomic Analyses Reveal Differential Regulation of Terpenoid Secondary Metabolites in *Hericium coralloides*. J. Fungi.

[34] Zhang Z.-F., Lv G.-Y., Song T.-T., Xu Z.-W., Wang M.-Y. (2024). Effects of different extraction methods on the structural and biological properties of *Hericium coralloides* polysaccharides. Food Chem..

[35] Wang X.Y., Zhang D.D., Yin J.Y., Nie S.P., Xie M.Y. (2019). Recent developments in *Hericium erinaceus* polysaccharides: Extraction, purification, structural characteristics and biological activities. Crit. Rev. Food Sci. Nutr..

[36] Wittstein K., Rascher M., Rupcic Z., Löwen E., Winter B., Köster R.W., Stadler M. (2016). Corallocins A-C, Nerve Growth and Brain-Derived Neurotrophic Factor Inducing Metabolites from the Mushroom *Hericium coralloides*. J. Nat. Prod..

[37] Ryu S.H., Hong S.M., Khan Z., Lee S.K., Vishwanath M., Turk A., Yeon S.W., Jo Y.H., Lee D.H., Lee J.K. (2021). Neurotrophic isoindolinones from the fruiting bodies of *Hericium erinaceus*. Bioorg. Med. Chem. Lett..

[38] Sum W.C., Gonkhom D., Ibrahim M.A.A., Stadler M., Ebada S.S. (2023). New isoindolinone derivatives isolated from the fruiting bodies of the basidiomycete *Hericium coralloides*. Mycol. Prog..

[39] Martínez-Mármol R., Chai Y., Conroy J.N., Khan Z., Hong S.-M., Kim S.B., Gormal R.S., Lee D.H., Lee J.K., Coulson E.J. (2023). Hericerin derivatives activates a pan-neurotrophic pathway in central hippocampal neurons converging to ERK1/2 signaling enhancing spatial memory. J. Neurochem..

[40] Wang K., Bao L., Qi Q., Zhao F., Ma K., Pei Y., Liu H. (2015). Erinacerins C-L, isoindolin-1-ones with α-glucosidase inhibitory activity from cultures of the medicinal mushroom *Hericium erinaceus*. J. Nat. Prod..

[41] Lazur J., Kała K., Krakowska A., Sułkowska-Ziaja K., Szewczyk A., Piotrowska J., Rospond B., Fidurski M., Marzec K., Muszyńska B. (2024). Analysis of bioactive substances and essential elements of mycelia and fruiting bodies of *Hericium* spp.. J. Food Compos. Anal..

[42] Franzoni F., Colognato R., Galetta F., Laurenza I., Barsotti M., Di Stefano R., Bocchetti R., Regoli F., Carpi A., Balbarini A. (2006). An In Vitro Study on the Free Radical Scavenging Capacity of Ergothioneine: Comparison with Reduced Glutathione, Uric Acid and Trolox. Biomed. Pharmacother.

[43] Li W., Zhou W., Kim E.J., Shim S.H., Kang H.K., Kim Y.H. (2015). Isolation and identification of aromatic compounds in Lion’s Mane Mushroom and their anticancer activities. Food Chem..

[44] Hu W., Song M., Wang C., Guo Z., Li Y., Wang D. (2021). Structural characterization of polysaccharide purified from *Hericium erinaceus* fermented mycelium and its pharmacological basis for application in Alzheimer’s disease: Oxidative stress related calcium homeostasis. Int. J. Biol. Macromol..

[45] Wu F., Zhou C., Zhou D., Ou S., Huang H. (2017). Structural characterization of a novel polysaccharide fraction from *Hericium erinaceus* and its signaling pathways involved in macrophage immunomodulatory activity. J. Funct. Foods.

[46] Li H., Feng J., Liu C., Hou S., Meng J., Liu J.Y., Zilong S., Chang M.C. (2024). Polysaccharides from an edible mushroom, *Hericium erinaceus*, alleviate ulcerative colitis in mice by inhibiting the NLRP3 inflammasomes and reestablish intestinal homeostasis. Int. J. Biol. Macromol..

[47] Lin J.Y., Chen Y.P., Lin T.W., Li T.J., Chen Y.W., Li I.C., Chen C.C. (2024). Discovery of a New Compound, Erinacerin W, from the Mycelia of *Hericium erinaceus*, with Immunomodulatory and Neuroprotective Effects. Molecules.

[48] Sum W.C., Ebada S.S., Kirchenwitz M., Kellner H., Ibrahim M.A.A., Stradal T.E.B., Matasyoh J.C., Stadler M. (2023). Hericioic Acids A-G and Hericiofuranoic Acid; Neurotrophic Agents from Cultures of the European Mushroom *Hericium flagellum*. J. Agric. Food Chem..

[49] Shimbo M., Kawagishi H., Yokogoshi H. (2005). Erinacine A increases catecholamine and nerve growth factor content in the central nervous system of rats. Nutr. Res..

[50] Xie L., Zhu G., Shang J., Chen X., Zhang C., Ji X., Wei Y. (2021). An overview on the biological activity and anti-cancer mechanism of lovastatin. Cell. Signal..

[51] Fu T.T., Shen L. (2022). Ergothioneine as a natural antioxidant against oxidative stress-related diseases. Front. Pharmacol..

[52] Li L.N., Wang L., Guo X.L. (2019). Chemical constituents from the culture of the fungus *Hericium alpestre*. J. Asian Nat. Prod. Res..

[53] Qi J., Wu J., Kang S., Gao J., Hirokazu K., Liu H., Liu C. (2024). The chemical structures, biosynthesis, and biological activities of secondary metabolites from the culinary-medicinal mushrooms of the genus *Hericium*: A review. Chin. J. Nat. Med..

[54] Yang J.Y., Tao L., Lou D., Patabendige N.M., Ediriweera A.N., Liu S., Lu W., Tarafder E., Rapior S., Hapuarachchi K.K. (2025). Innovative applications of medicinal mushrooms in functional foods and nutraceuticals: A focus on health-boosting beverages. Front. Cell. Infect. Microbiol..

[55] SPb ART-LAJF. https://www.spb-artlife.ru/art-layf---sorbiotik-250-g/.

[56] Ozon. https://www.ozon.ru/product/ezhovik-korallovidnyy-700mg-molotyy-ezhevik-mitseliy-lions-mane-mikrodozing-1639925127/?tab=reviews&__rr=1&abt_att=1.

[57] Mil’Fei. https://milfey-shop.ru/nativitan-nativitan-24.

[58] Ozon. https://www.ozon.ru/product/kontsentrat-pishchevoy-na-osnove-rastitelnogo-syrya-batel-fungosbor-stimuliruyushchiy-pri-1315807345/?at=79tn7Yw8Yf4wWgLkugN3lo7Fkz6rxRfrZzy63H0Kq4rW.

[59] Ozon. https://www.ozon.ru/product/ezhovik-grebenchatyy-ezhevik-korallovidnyy-izviliny-mitseliy-100-gramm-1761031816/?at=Brtz2NywNtrXJ7jLTE5vNjOfn23XlyS96XPY0i017v5q.

[60] Fang X., Song J., Zhou K., Zi X., Sun B., Bao H., Li L. (2023). Molecular Mechanism Pathways of Natural Compounds for the Treatment of Non-Alcoholic Fatty Liver Disease. Molecules.

[61] Feng J., Li M.H., Yao T.T., Yi X.J., Gao H.N. (2025). Research progress on AMPK in the pathogenesis and treatment of MASLD. Front. Immunol..

[62] Liu W., Zhang Y., Zheng M., Ye Y., Shi M., Wang X., Cao L., Wang L. (2024). Polysaccharides in Medicinal and Food Homologous Plants regulate intestinal flora to improve type 2 diabetes: Systematic review. Phytomed. Int. J. Phytother. Phytopharm..

[63] Nassir F. (2022). NAFLD: Mechanisms, Treatments, and Biomarkers. Biomolecules.

[64] Liu Q., Luan H., Duan Z., Ai J., Wang Y., Chen P. (2025). Efficacy of flavonoids in non-alcoholic fatty liver disease: An updated systematic review and meta-analysis. Front. Nutr..

[65] Ezhilarasan D., Lakshmi T. (2022). A Molecular Insight into the Role of Antioxidants in Nonalcoholic Fatty Liver Diseases. Oxidative Med. Cell. Longev..

[66] Lu H., Yang S., Li W., Zheng B., Zeng S., Chen H. (2025). *Hericium erinaceus* Protein Alleviates High-Fat Diet-Induced Hepatic Lipid Accumulation and Oxidative Stress In Vivo. Foods.

[67] Zhang C., Shi J., Shi L. (2025). Natural products intervene in non-alcoholic fatty liver disease by regulating the AMPK signaling pathway: Preclinical evidence and mechanism. Front. Pharmacol..

[68] Eslami L., Merat S., Malekzadeh R., Nasseri-Moghaddam S., Aramin H. (2013). Statins for non-alcoholic fatty liver disease and non-alcoholic steatohepatitis. Cochrane Database Syst. Rev..

[69] Nowis D., Malenda A., Furs K., Oleszczak B., Sadowski R., Chlebowska J., Firczuk M., Bujnicki J.M., Staruch A.D., Zagozdzon R. (2014). Statins impair glucose uptake in human cells. BMJ Open Diabetes Res. Care.

[70] Dolivo D.M., Reed C.R., Gargiulo K.A., Rodrigues A.E., Galiano R.D., Mustoe T.A., Hong S.J. (2023). Anti-fibrotic effects of statin drugs: A review of evidence and mechanisms. Biochem. Pharmacol..

[71] Lv X., Nie C., Shi Y., Qiao Q., Gao J., Zou Y., Yang J., Chen L., Hou X. (2024). Ergothioneine ameliorates metabolic dysfunction-Associated Steatotic Liver Disease (MASLD) by enhancing autophagy, inhibiting oxidative damage and inflammation. Lipids Health Dis..

[72] Cheah I.K., Tang R., Ye P., Yew T.S., Lim K.H., Halliwell B. (2016). Liver ergothioneine accumulation in a guinea pig model of non-alcoholic fatty liver disease. A possible mechanism of defence?. Free Radic. Res..

[73] Contato A.G., Conte-Junior C.A. (2025). Lion’s Mane Mushroom (*Hericium erinaceus*): A Neuroprotective Fungus with Antioxidant, Anti-Inflammatory, and Antimicrobial Potential—A Narrative Review. Nutrients.

